# Supplementary material for: Glucose-oxidase like catalytic mechanism of noble metal nanozymes
Source: Nat Commun. 2021 Jun 7;12:3375. doi: 10.1038/s41467-021-23737-1 (PMC8184917; doi:10.1038/s41467-021-23737-1)
Supplement: Supplementary file 1 — SUPPLEMENTARY INFO [file 41467_2021_23737_MOESM1_ESM.pdf]

Supplementary Information

Glucose-oxidase like catalytic mechanism of noble metal nanozymes

Jinxing Chen,[1,2] Qian Ma,[1,2] Minghua Li,[1] Daiyong Chao,[1] Liang Huang,[1,2] Weiwei Wu,[1,2] Youxing Fang,[*1] and Shaojun Dong,[*1,2]

1 State Key Laboratory of Electroanalytical Chemistry, Changchun Institute of Applied Chemistry, Chinese Academy of Sciences, Changchun, Jilin 130022, PR China
2 University of Science and Technology of China, Hefei, Anhui 230026, PR China

Email: Youxing Fang, fangyx@ciac.ac.cn; Shaojun Dong, dongsj@ciac.ac.cn.

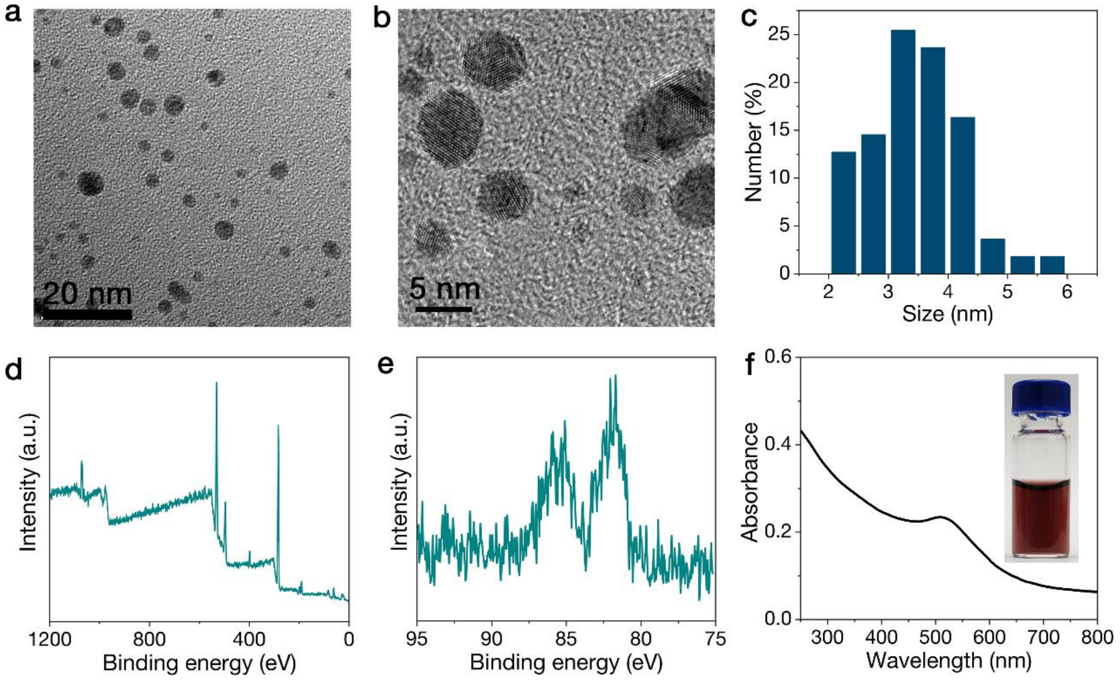

Supplementary Fig. 1. **Characterizations of Au nanoparticles.** (a, b) TEM images of Au nanoparticles. (c) Size distribution of Au nanoparticles. (d, e) XPS spectra of Au nanoparticles. (f) UV-Vis spectrum of Au nanoparticles. The inset shows Au nanoparticles dispersed in water.

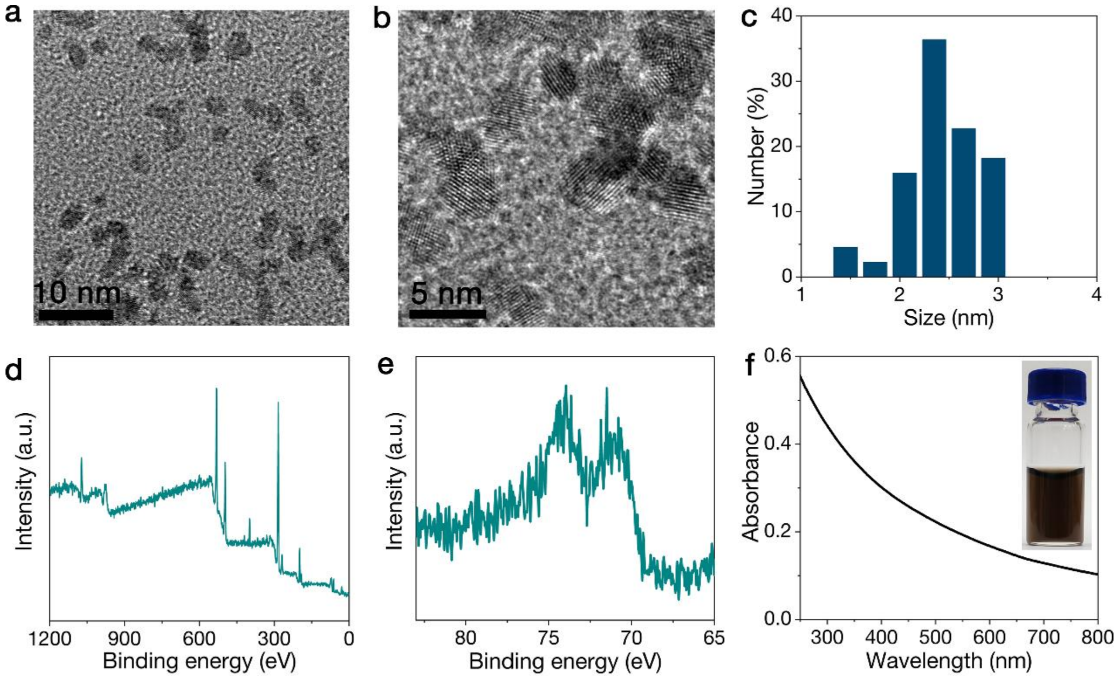

Supplementary Fig. 2. Characterizations of Pt nanoparticles. (a, b) TEM images of Pt nanoparticles. (c) Size distribution of Pt nanoparticles. (d, e) XPS spectra of Pt nanoparticles. (f) UV-Vis spectrum of Pt nanoparticles. The inset shows Pt nanoparticles dispersed in water.

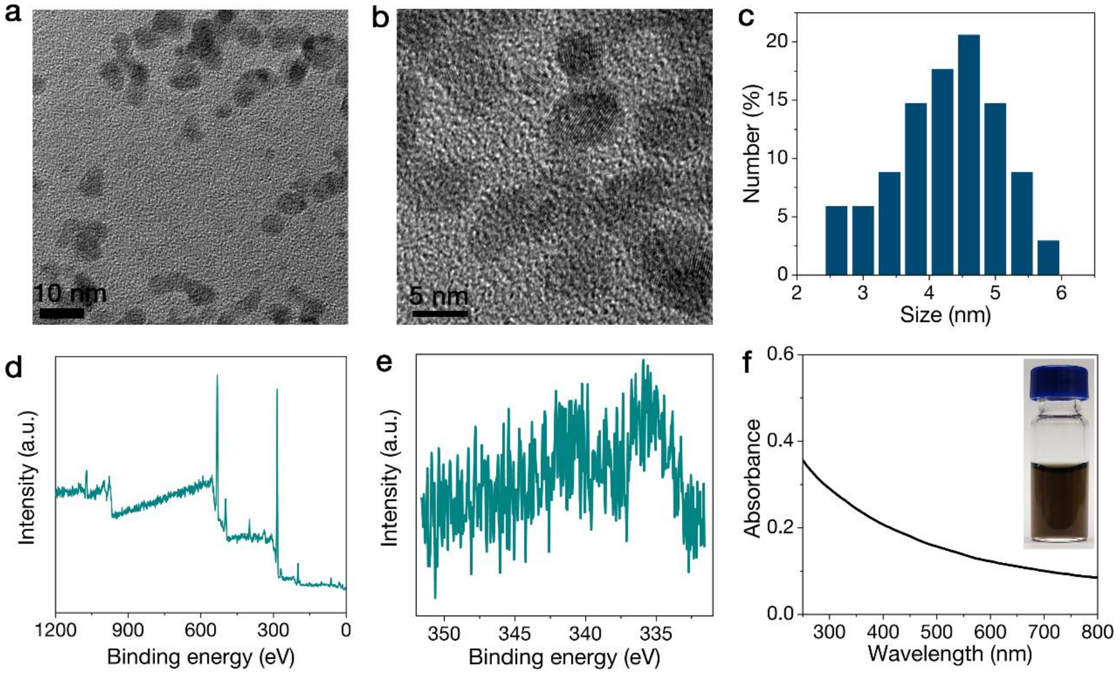

Supplementary Fig. 3. Characterizations of Pd nanoparticles. (a, b) TEM images of Pd nanoparticles. (c) Size distribution of Pd nanoparticles. (d, e) XPS spectra of Pd nanoparticles. (f) UV-Vis spectrum of Pd nanoparticles. The inset shows Pd nanoparticles dispersed in water.

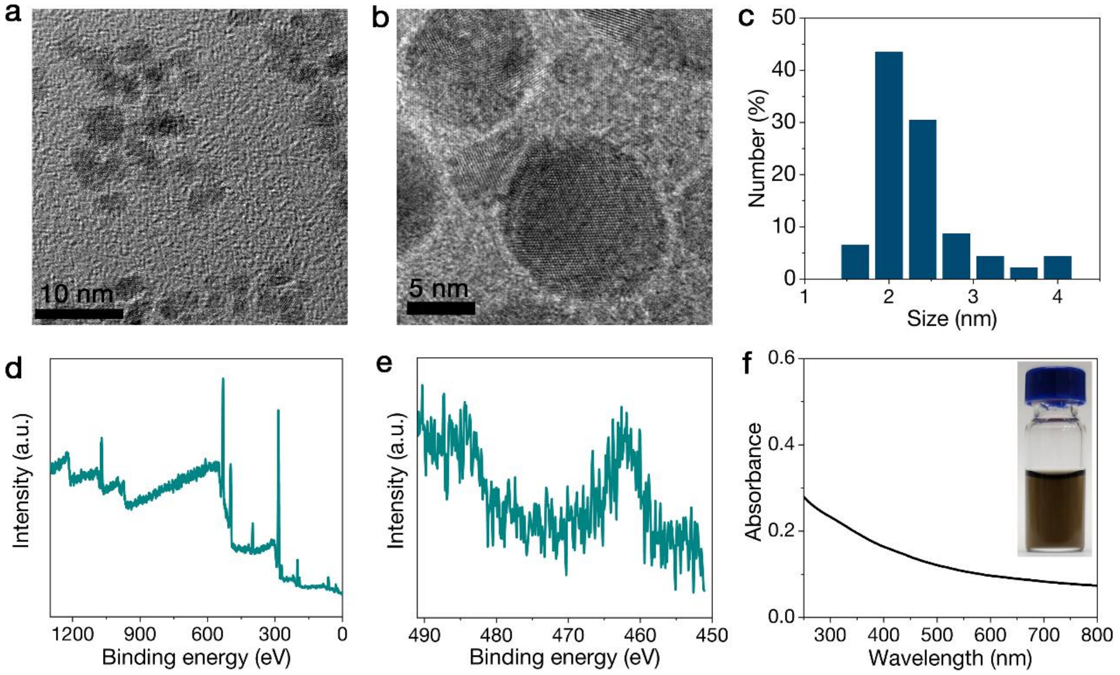

Supplementary Fig. 4. Characterizations of Ru nanoparticles. (a, b) TEM images of Ru nanoparticles. (c) Size distribution of Ru nanoparticles. (d, e) XPS spectra of Ru nanoparticles. (f) UV-Vis spectrum of Ru nanoparticles. The inset shows Ru nanoparticles dispersed in water.

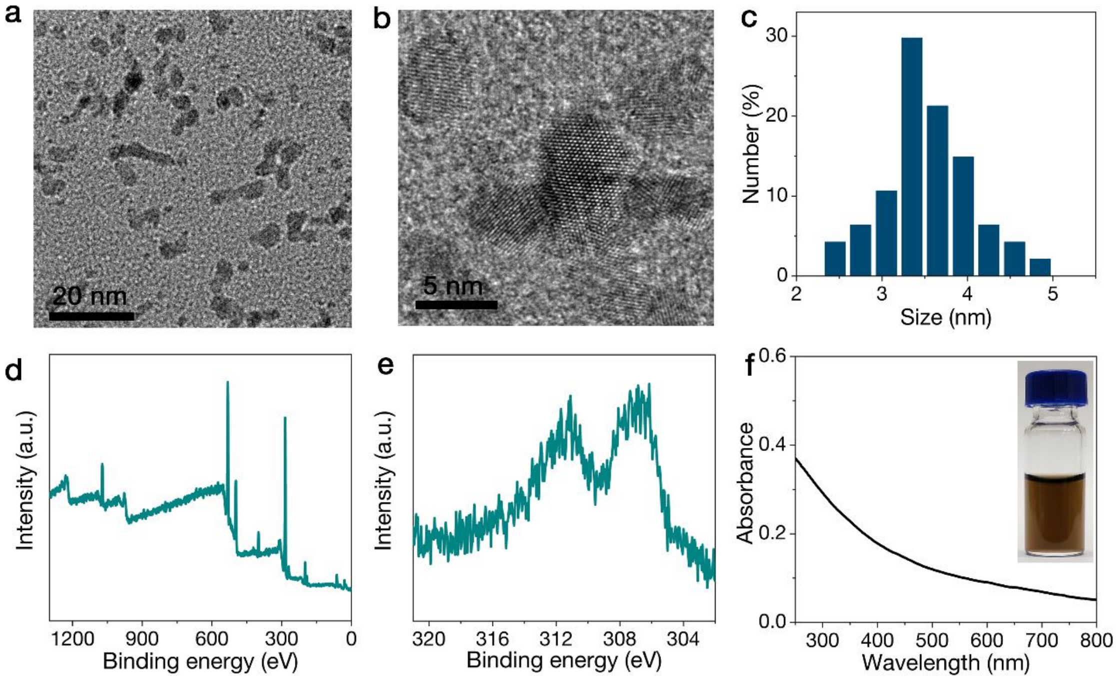

Supplementary Fig. 5. Characterizations of Rh nanoparticles. (a, b) TEM images of Rh nanoparticles. (c) Size distribution of Rh nanoparticles. (d, e) XPS spectra of Rh nanoparticles. (f) UV-Vis spectrum of Rh nanoparticles. The inset shows Rh nanoparticles dispersed in water.

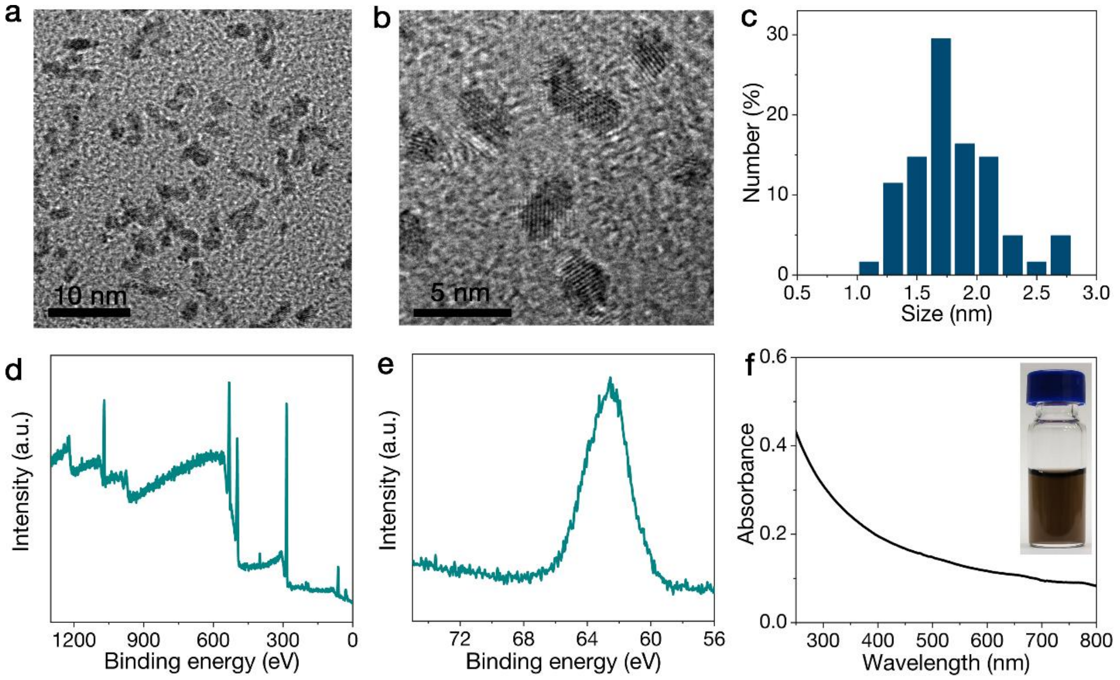

Supplementary Fig. 6. Characterizations of Ir nanoparticles. (a, b) TEM images of Ir nanoparticles. (c) Size distribution of Ir nanoparticles. (d, e) XPS spectra of Ir nanoparticles. (f) UV-Vis spectrum of Ir nanoparticles. The inset shows Ir nanoparticles dispersed in water.

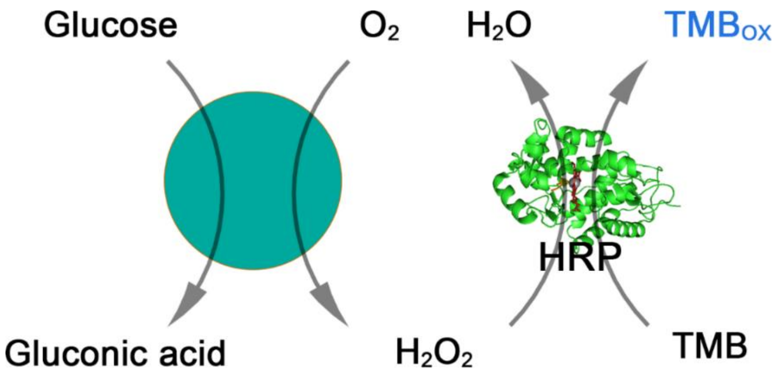

Supplementary Fig. 7. Schematic representation of the detection of H_2O_2 produced during oxidation of glucose catalyzed by Au NPs.

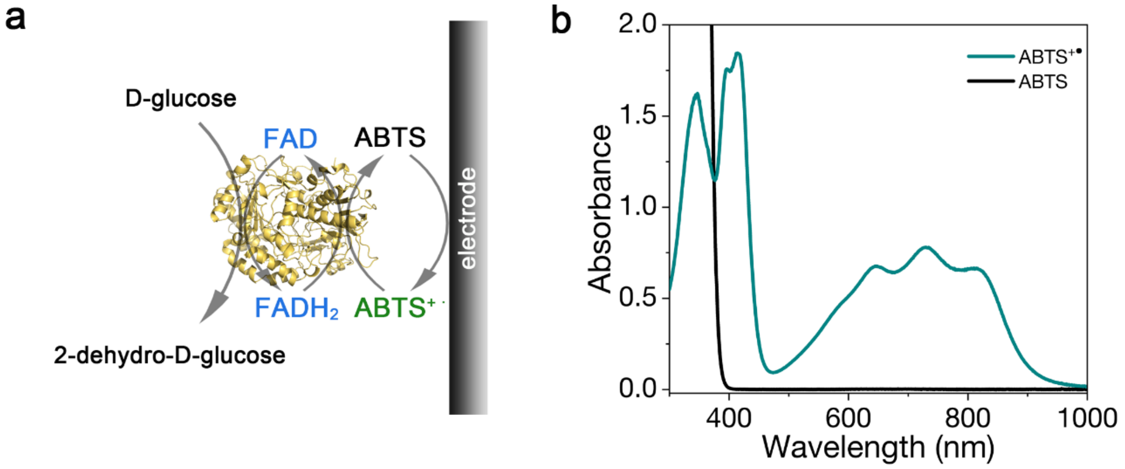

Supplementary Fig. 8. (a) Schematic diagram of ABTS as a mediator of glucose oxidase. (b) UV-vis spectra of ABTS and $ABTS^{+\bullet}$.

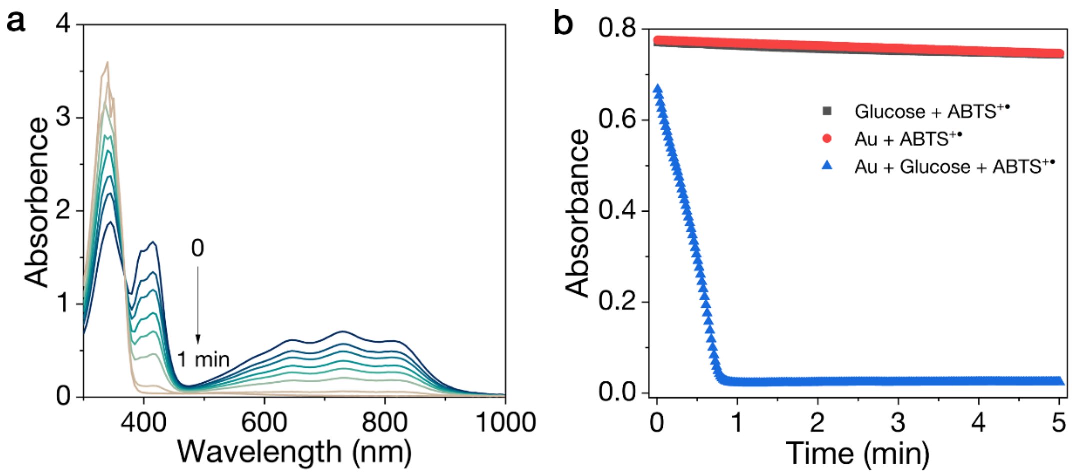

Supplementary Fig. 9. Catalytic reduction of $ABTS^{+\bullet}$ by Au NPs. (a) UV-vis spectra of $ABTS^{+\bullet}$ over time in the presence of Au and glucose. (b) The absorption changes of $ABTS^{+\bullet}$ at 734 nm.

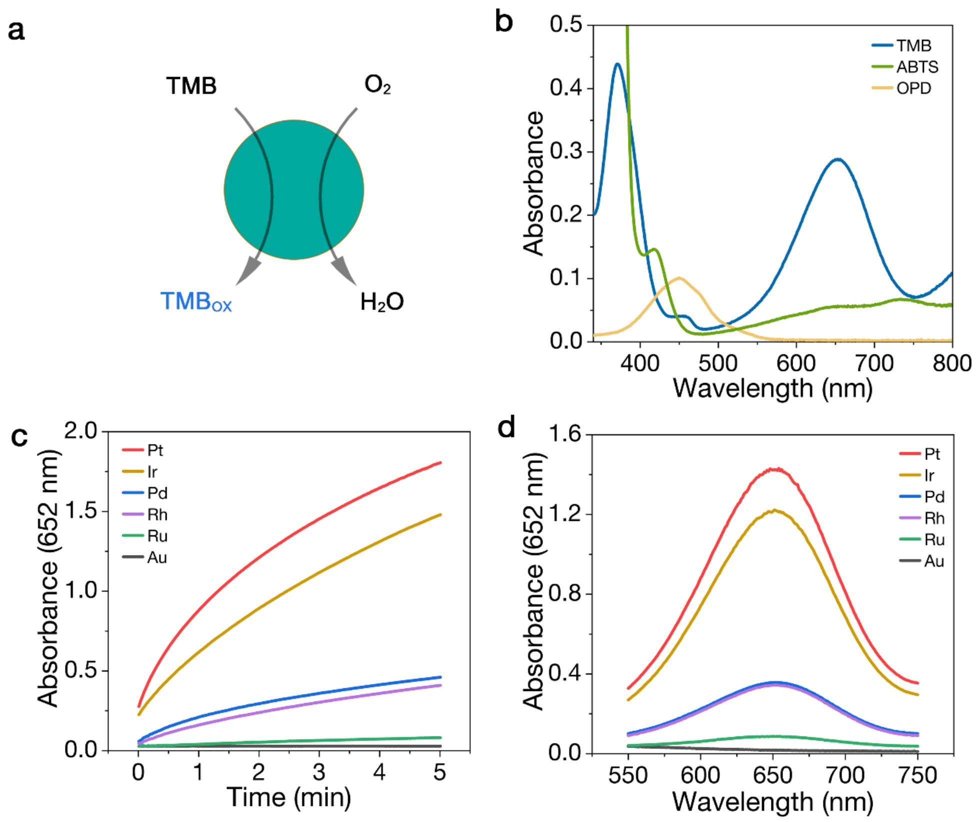

Supplementary Fig. 10. Oxidase like activities of noble metal nanozymes. (a) Schematic representation of the oxidase like activity. (b) UV-vis spectra of TMB, OPD, and ABTS after oxidation. (c) Time dependent absorption changes of TMB at 652 nm in the presence of different catalysts. (d) UV-vis spectra of TMB in the presence of different catalysts.

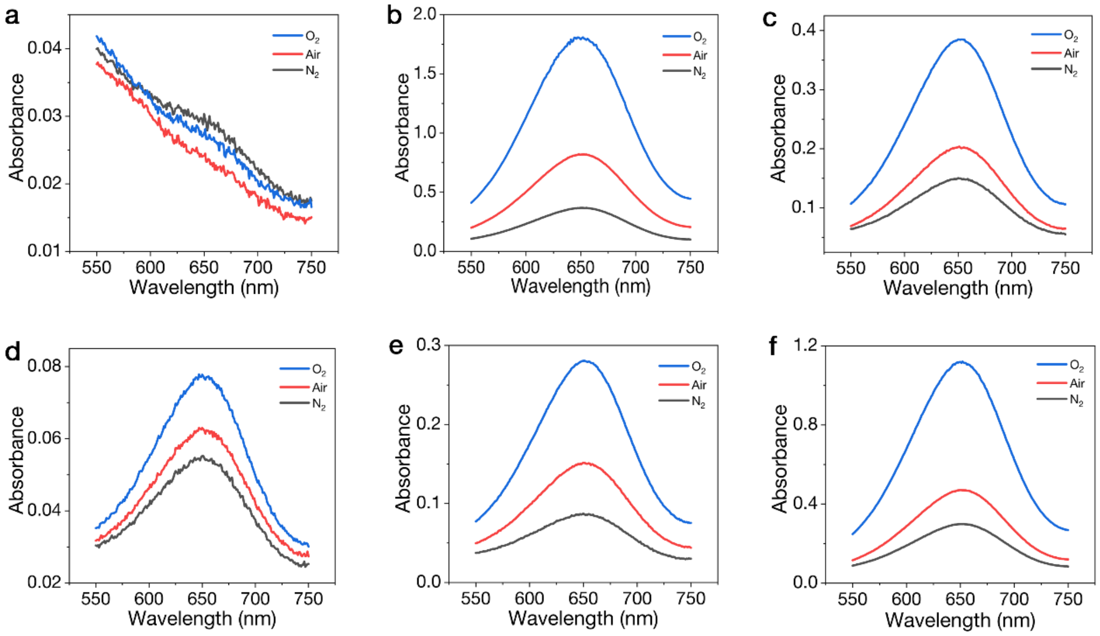

Supplementary Fig. 11. Oxidase like activities of noble metal nanozymes.UV-vis spectra of TMB in the presence of (a) Au, (b) Pt, (c) Pd, (d) Ru, (e) Rh, and (f) Ir in different gas saturated solutions.

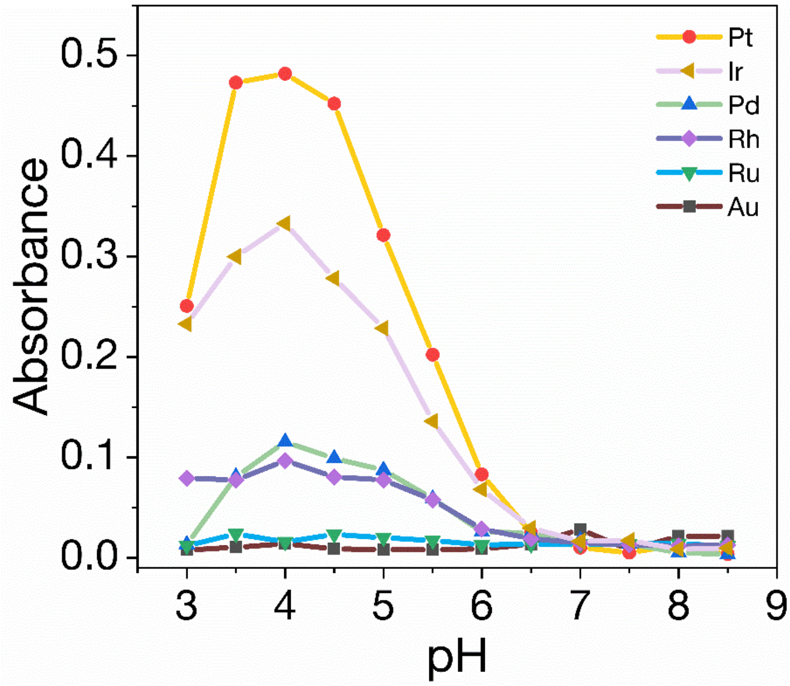

Supplementary Fig. 12. The pH dependent oxidase-mimic activities of different catalysts.

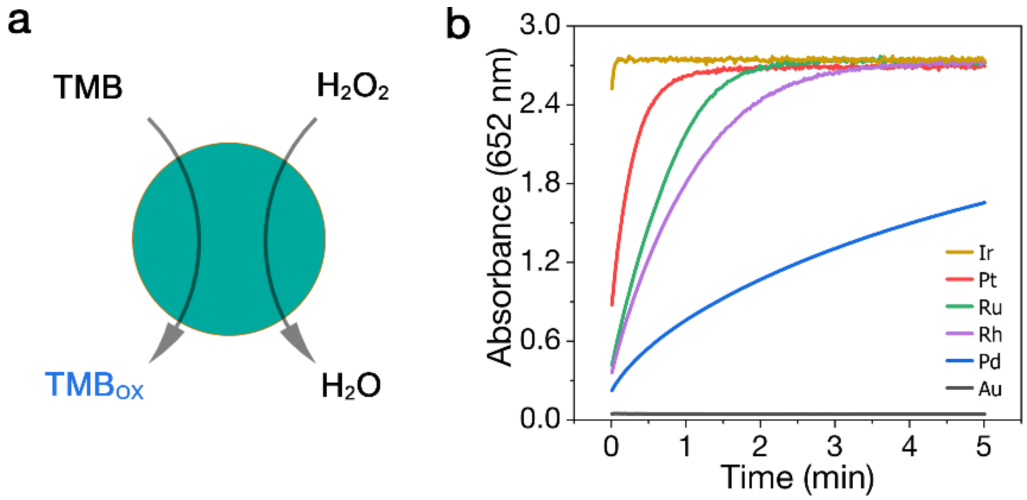

Supplementary Fig. 13. Peroxidase like activities of noble metal nanozymes. (a) Schematic representation of the peroxidase like activity. (b) Time dependent absorption changes of TMB at 652 nm in the presence of H_2O_2 and different catalysts.

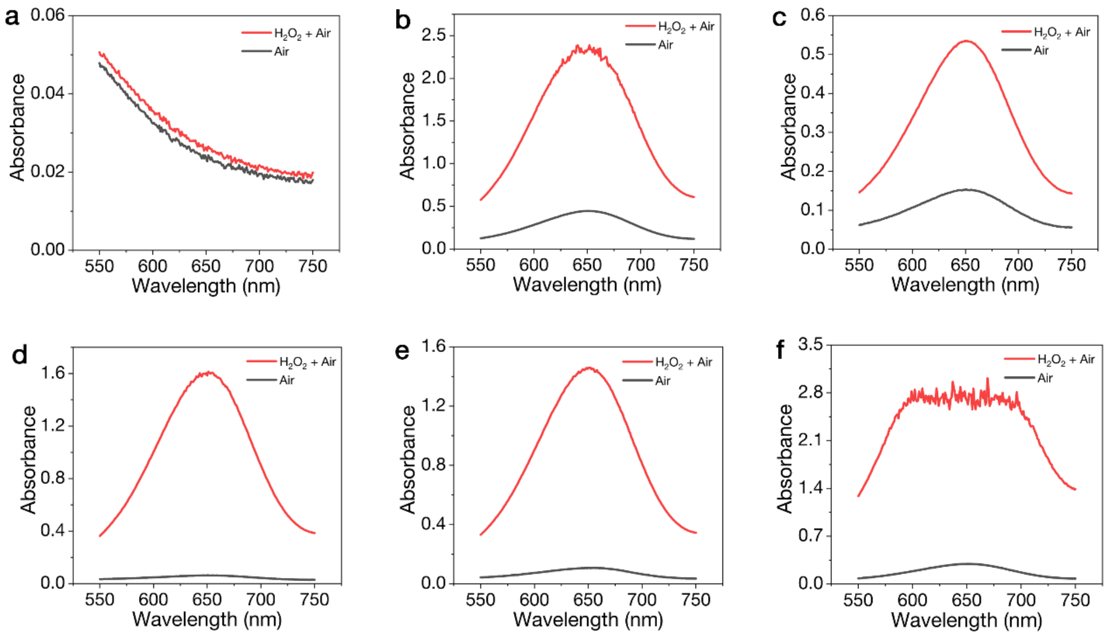

Supplementary Fig. 14. Peroxidase like activities of noble metal nanozymes. UV-vis spectra of TMB in the presence of (a) Au, (b) Pt, (c) Pd, (d) Ru, (e) Rh, (f) Ir under different conditions.

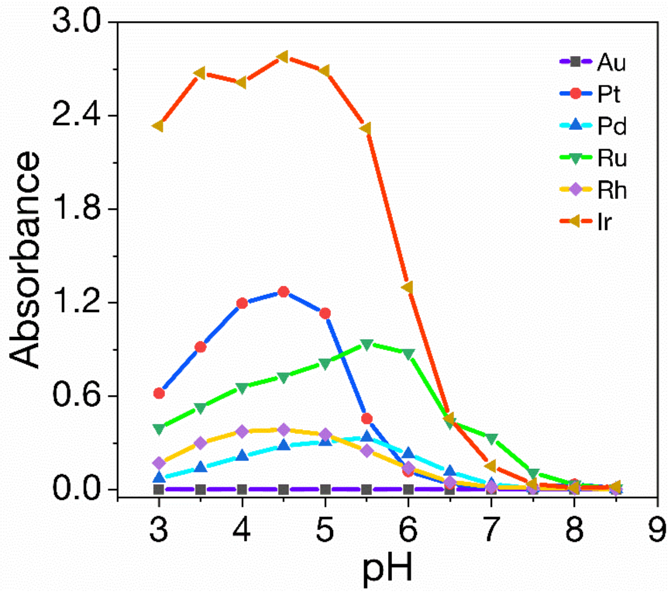

Supplementary Fig. 15. The pH dependent peroxidase-mimic activities of different catalysts.

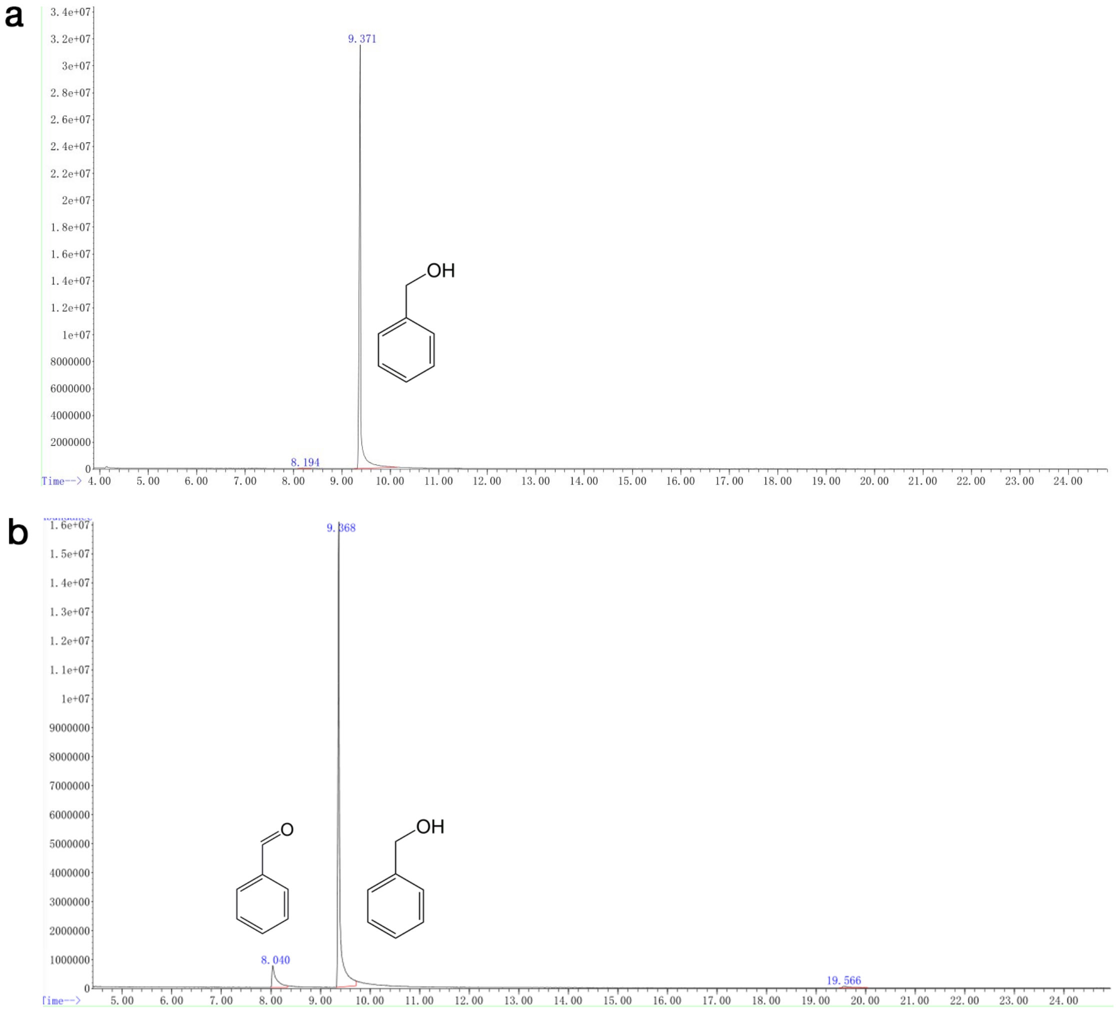

Supplementary Fig. 16. Chromatogram of benzyl alcohol before (a) and after (b) Au catalyzed oxidation reaction.

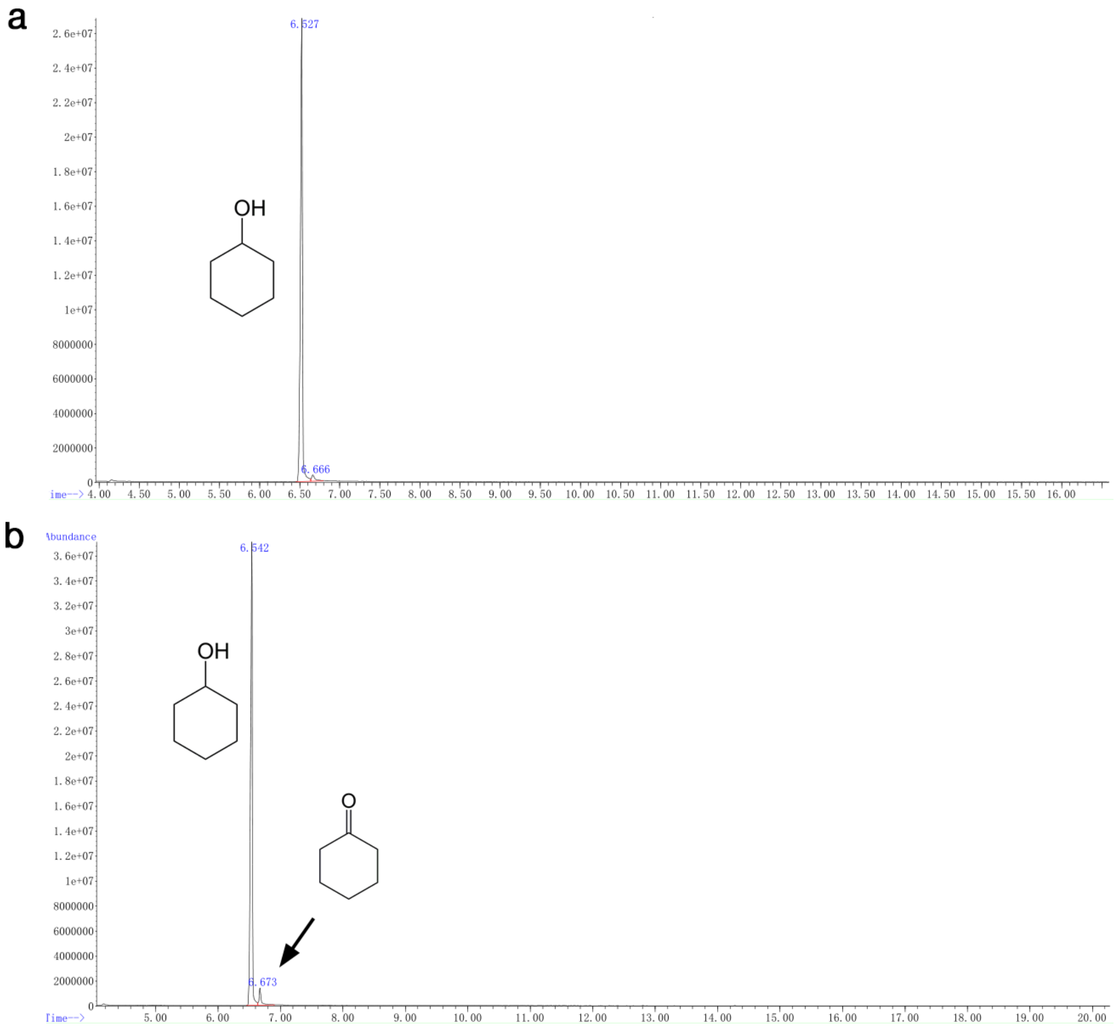

Supplementary Fig. 17. Chromatogram of cyclohexanol before (a) and after (b) Au catalyzed oxidation reaction.

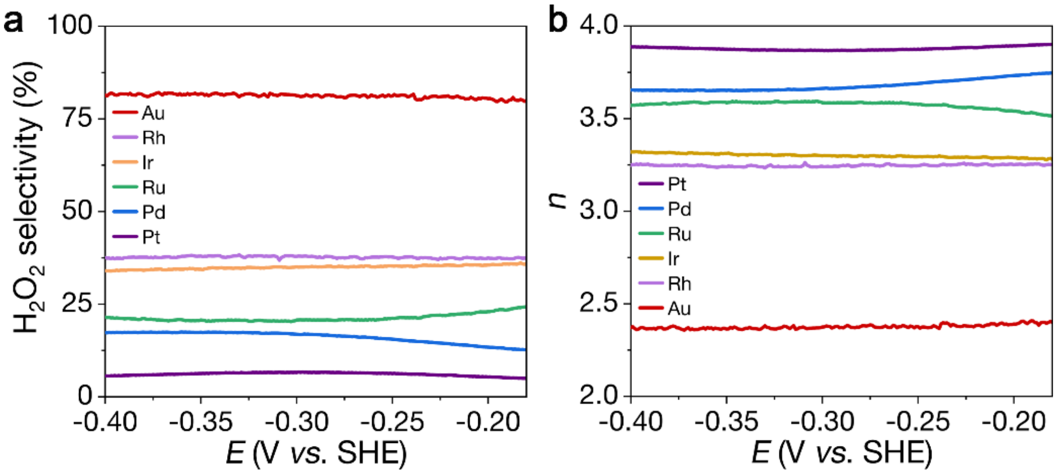

Supplementary Fig. 18. ORR performance. (a) O_2 reduction selectivity toward H_2O_2 production and the calculated values of electron transfer number.

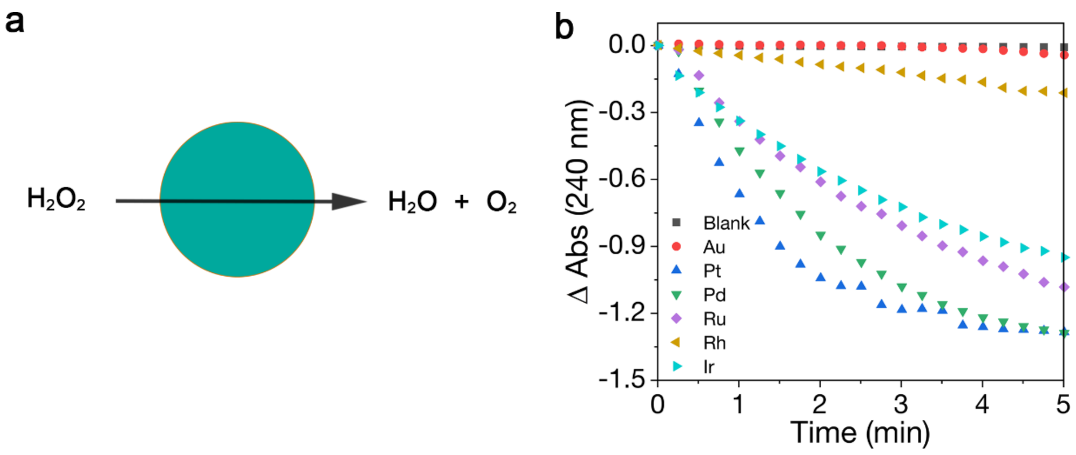

Supplementary Fig. 19. Catalase like activities of noble metal nanozymes. (a) Schematic representation of the catalase like activity. (b) Time dependent absorption changes of H_2O_2 at 240 nm in the presence of different catalysts.

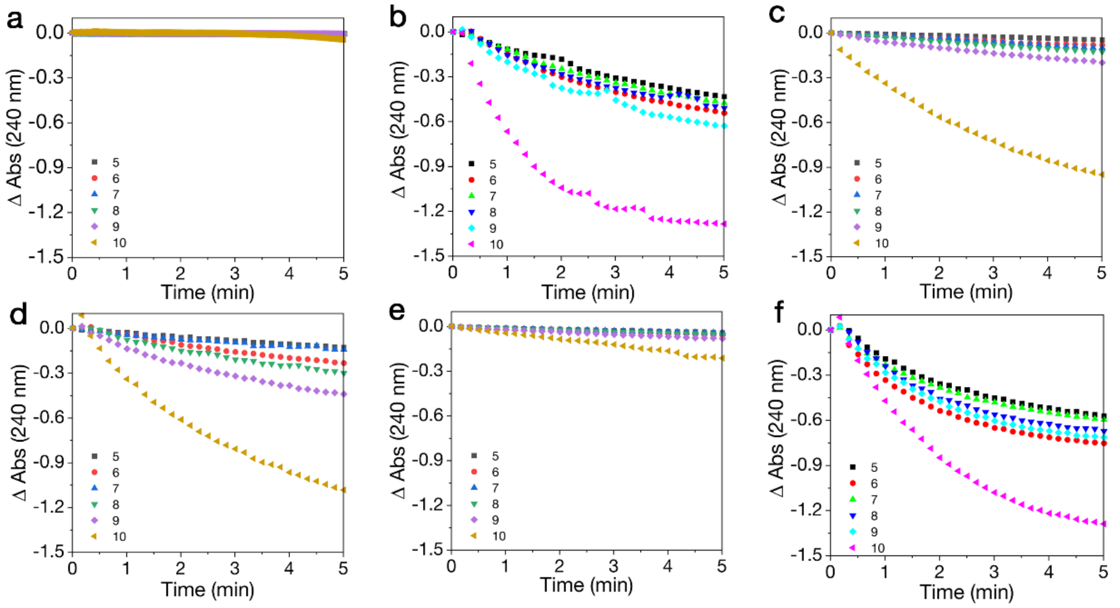

Supplementary Fig. 20. Catalase like activities of noble metal nanozymes. Time dependent absorption changes of H_2O_2 at 240 nm in the presence of (a) Au, (b) Pt, (c) Pd, (d) Ru, (e) Rh, (f) Ir in buffer solutions at different pH values.

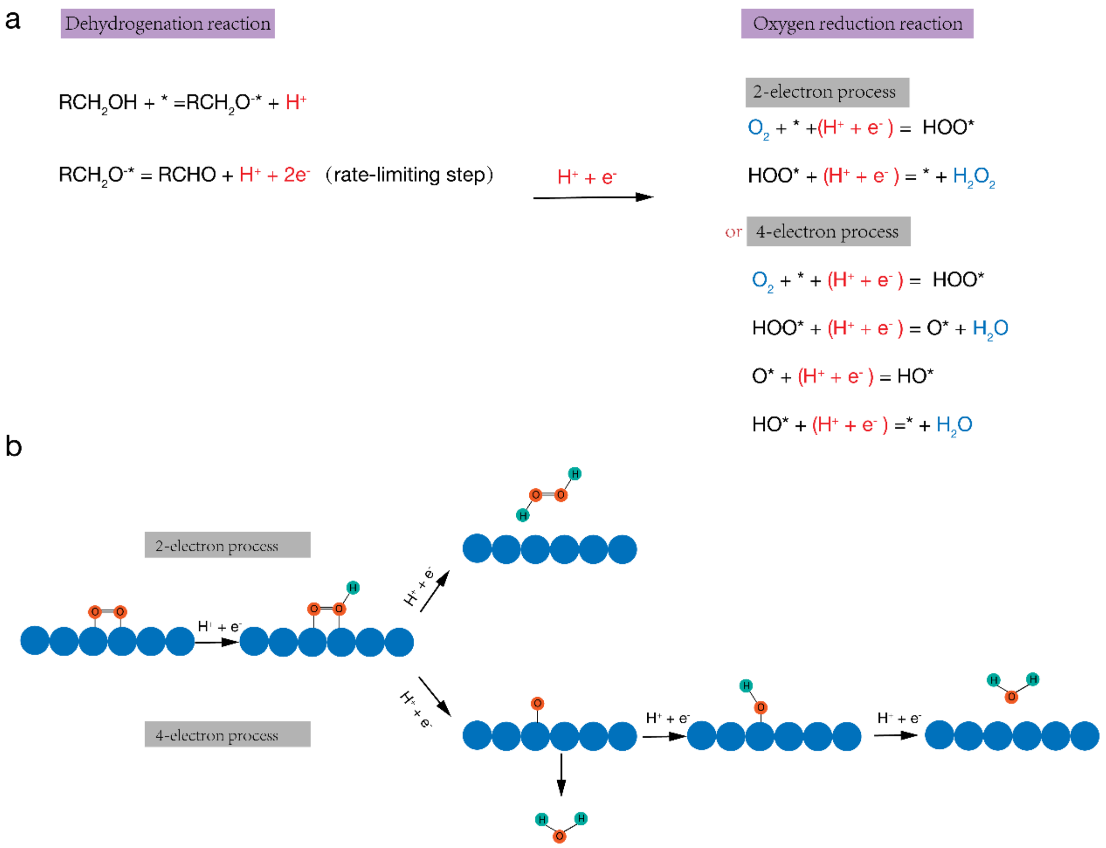

Supplementary Fig. 21. Proposed mechanism of glucose oxidation catalyzed by noble metal NPs. (a) Mechanism of glucose oxidation catalyzed by noble metal NPs. (b) The difference between 2e- path and 4e- path in oxygen reduction reaction. * denote the active sites at the surface of NPs

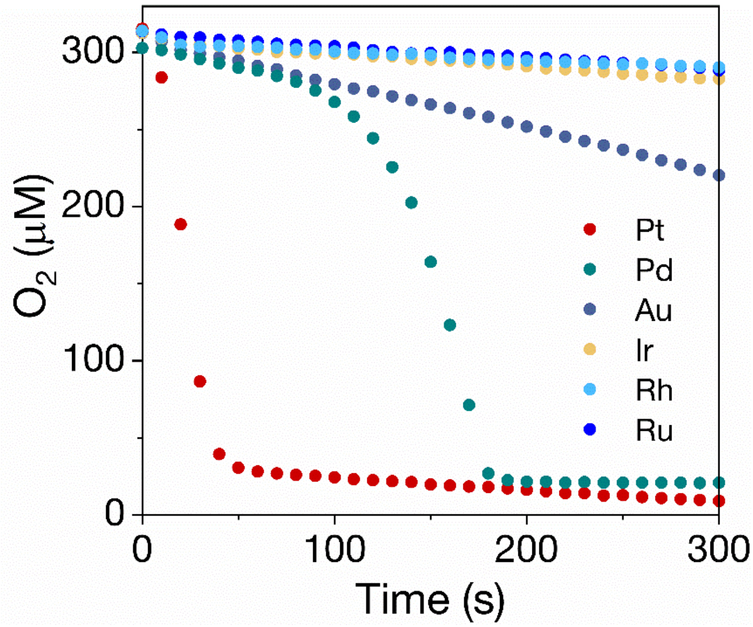

Supplementary Fig. 22. Oxygen-consumption assays. O_2 concentrations over time in the presence of HCOOK and different catalysts.

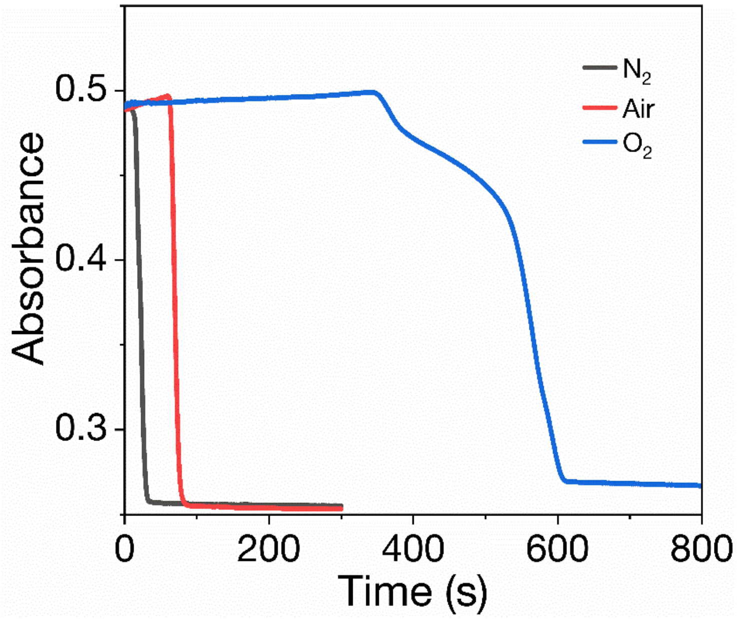

Supplementary Fig. 23. FMN reduction. UV-visible absorption at 450 nm of the mixed solutions containing FMN, HCOOK, and Pt in N_2, Air, and O_2-saturated buffer.

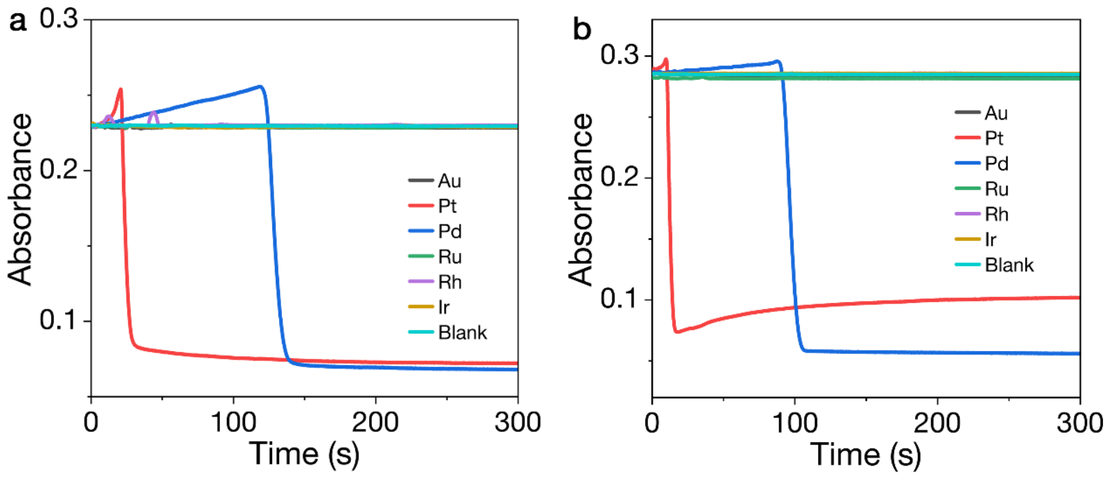

Supplementary Fig. 24. FAD reduction. (a) UV-visible absorption of FAD at 450 nm in the presence of HCOOK and different catalysts. (b) UV-visible absorptions of FMN at 450 nm in the presence of HCOOK and different catalysts.

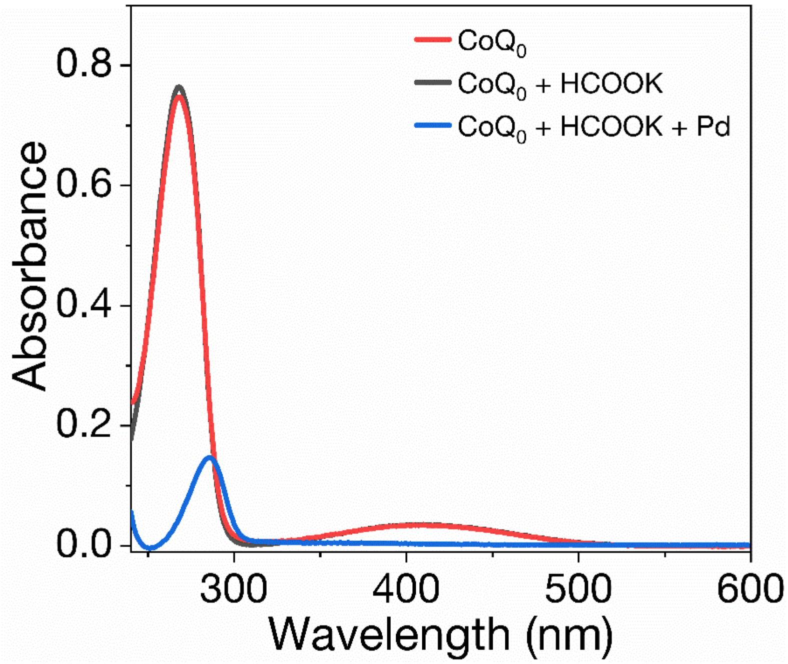

Supplementary Fig. 25. CoQ_0 reduction. UV-visible absorption spectra of CoQ_0, CoQ_0 + HCOOK, and CoQ_0 + HCOOK + Pd after reacting for 5 min.

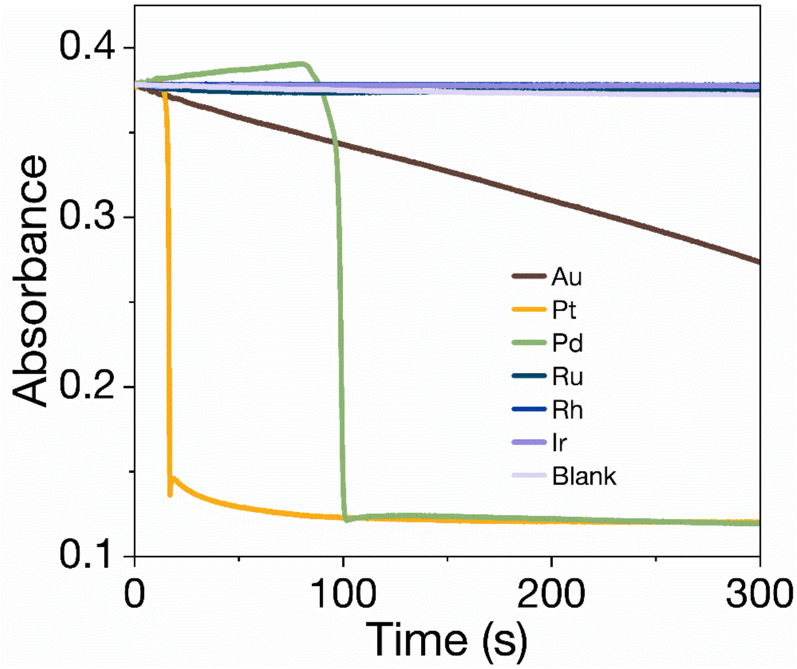

Supplementary Fig. 26. CoQ_0 reduction. UV-visible absorptions of CoQ_0 at 268 nm in the presence of HCOOK and different catalysts.

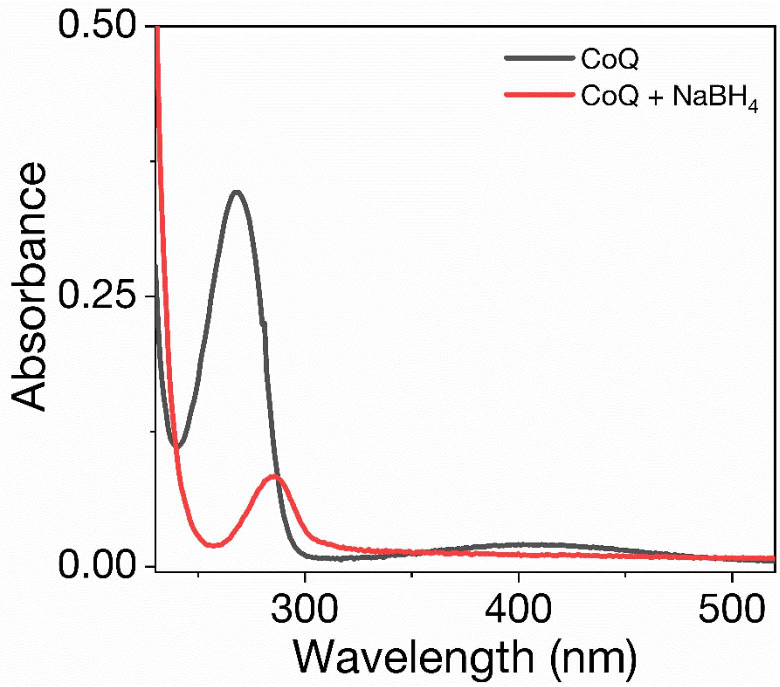

Supplementary Fig. 27. CoQ_0 reduction. UV-visible absorption spectra of CoQ_0, CoQ_0 + $NaBH_4$. Since $NaBH_4$ can reduce CoQ_0 rapidly, we have not measured the catalytic effect of nanoparticles on this reaction.

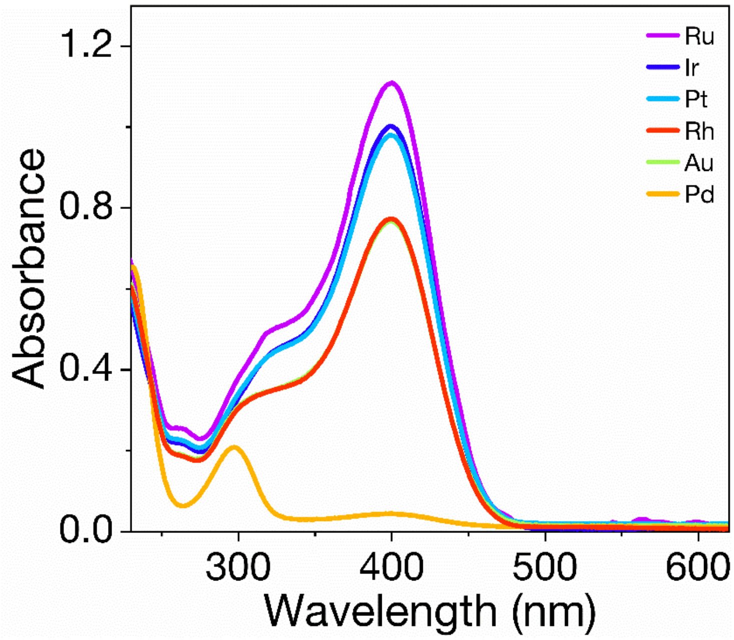

Supplementary Fig. 28. pNP reduction.UV-visible absorption spectra of pNP in the presence of $NaBH_4$ and different catalysts.
